# A randomized controlled trial of long term effect of BCM guided fluid management in MHD patients (BOCOMO study): rationales and study design

**DOI:** 10.1186/1471-2369-13-120

**Published:** 2012-09-25

**Authors:** Li Liu, Gang Long, Jianwei Ren, Jijun Li, Jinsheng Xu, Jinghong Lei, Mao Li, Moyan Qiu, Ping Yuan, Weiming Sun, Shan Lin, Wenjun Liu, Yi Sun, Yingchun Ma, Yonghui Mao, Yulan Shen, Li Zuo

**Affiliations:** 1 Institute of Nephrology, Peking University First Hospital, Beijing, China; 2 Renal Division, Peking University First Hospital, Beijing, China; 3 Renal Department, PLA General Hospital First Hospital, Beijing, China; 4 Renal Department, Hebei Medical University Forth Hospital, Hebei, China; 5 Renal Department, Beijing Aerospace General Hospital, Beijing, China; 6 Renal Department, Beijing Puren Hospital, Beijing, China; 7 Renal Department, Beijing Wangjing Hospital, Beijing, China; 8 Renal Department, Tianjin Third Central Hospital, Tianjin, China; 9 Renal Department, Beijing Shijitan Hospital, Beijing, China; 10 Renal Department, Tianjin Medical University General Hospital, Tianjin, China; 11 Renal Department, Guanganmen Hospital, China Academy of Chinese Medical Sciences, Guanganmen, China; 12 Renal Department, Capital University Fuxing Hospital, Beijing, China; 13 Renal Department, China Rehabilitation Research Center, Beijing Boai Hospital, Beijing, China; 14 Renal Department, Beijing Hospital of Ministry of Health, Beijing, China; 15 Renal Department, Miyun Hospital, Beijing, China; 16 Institute of Nephrology, Peking University First Hospital, Beijing, China; 17 Renal Division, Peking University First Hospital, Institute of Nephrology, Peking University, 8 Xishiku Street, Xicheng District, Beijing, 100034, People's Republic of China

**Keywords:** Hemodialysis, Bioimpedance, Dry weight, Body composition monitor, Randomized controlled trial

## Abstract

**Background:**

Bioimpedance analysis (BIA) has been reported as helpful in identifying hypervolemia. Observation data showed that hypervolemic maintenance hemodialysis (MHD) patients identified using BIA methods have higher mortality risk. However, it is not known if BIA-guided fluid management can improve MHD patients’ survival. The objectives of the BOCOMO study are to evaluate the outcome of BIA guided fluid management compared with standard care.

**Methods:**

This is a multicenter, prospective, randomized, controlled trial. More than 1300 participants from 16 clinical sites will be included in the study. The enrolment period will last 6 months, and minimum length of follow-up will be 36 months. MHD patients aged between 18 years and 80 years who have been on MHD for at least 3 months and meet eligibility criteria will be invited to participate in the study. Participants will be randomized to BIA arm or control arm in a 1:1 ratio. A portable whole body bioimpedance spectroscopy device (BCM—Fresenius Medical Care D GmbH) will be used for BIA measurement at baseline for both arms of the study. In the BIA arm, additional BCM measurements will be performed every 2 months. The primary intent-to-treat analysis will compare outcomes for a composite endpoint of death, acute myocardial infarction, stroke or incident peripheral arterial occlusive disease between groups. Secondary endpoints will include left ventricular wall thickness, blood pressure, medications, and incidence and length of hospitalization.

**Discussions:**

Previous results regarding the benefit of strict fluid control are conflicting due to small sample sizes and unstable dry weight estimating methods. To our knowledge this is the first large-scale, multicentre, prospective, randomized controlled trial to assess whether BIS-guided volume management improves outcomes of MHD patients. The endpoints of the BOCOMO study are of utmost importance to health care providers. In order to obtain that aim, the study was designed with very careful important considerations related to the endpoints, sample size, inclusion criteria, exclusion criteria and so on. For example, annual mortality of Beijing MHD patients was around 10%. To reach statistical significance, the sample size will be very large. By using composite endpoint, the sample size becomes reasonable and feasible. Limiting inclusion to patients with urine volume less than 800 ml/day the day before dialysis session will limit confounding due to residual renal function effects on the measured parameters. Patients who had received BIS measurement within 3 months prior to enrolment are excluded as data from such measurements might lead to protocol violation. Although not all patients enrolled will be incident patients, we will record the vintage of dialysis in the multivariable analysis.

**Trial registration:**

Current Controlled Trials NCT01509937

## Background

Intra-dialytic symptoms are common among patients on maintenance hemodialysis (MHD). Adverse symptoms are associated with both hypervolemia and hypovolemia. Persistent hypervolemia causes hypertension, pulmonary edema, and congestive heart failure, and leads to higher mortality [1]. Compared with euvolemic patients, patients with recurrent episodes of intra-dialytic hypovolemia are at high risk of accelerated loss of residual renal function [2], vascular access function loss [3], brain atrophy [4], mesenteric infarction [5], and hence, higher morbidity and mortality [6,7].

Patients with high pre-dialysis (pre-HD) fluid overload experience higher mortality risk compared with those with less pre-HD fluid burden [1,8,9]. It is an important strategy to reduce pre-HD fluid burden by restricting inter-dialysis weight gain and to achieve euvolemic (dry) weight post-HD by appropriate ultrafiltration. At present there is no objective gold standard method for estimating target post-HD dry weight and clinical examination is unreliable.

Several observational studies showed that strict post-HD weight control was associated with better short term outcome [10] or long term survival [11], but other studies associated strict fluid control with increased morbidity and / or hospitalization [12,13]. These conflicting results regarding the relationship between fluid control and outcome mainly result from the lack of reliable method for the assessment of individual euvolemia with different studies used different definitions of dry weight including un-tolerated blood volume decrease or ultrafiltration limit [14].

Bioimpedance spectroscopy (BIS) had long been used to assess human body composition and has been validated by isotope dilution methods [15,16] and reference body composition methods [16-18]. Recently, Wizemann et al. [1] used BIS to assess MHD patients’ fluid status, and found that patients who had 15% or more expansion of extra-cellular fluid (ECF) suffered higher mortality risk compared with those had less than 15% ECF expansion.

BIS appears to be a promising and a valuable tool in aiding dry weight estimation for MHD patients. Although detailed BIS-based dry weight estimating methods were published [10,19-21], and observational studies showed the potential benefits of BIS [1,8], it is still not clear whether long-term outcome could be improved by regular dry weight adjustment according to BIS based method.

We designed this multicenter, prospective, randomized and controlled trial to explore the effect of BIS guided fluid management on long-term outcome in MHD patients.

## Methods

### Study design

This is an open label, multicenter, prospective, randomized controlled trial. The aim of the study is to assess if BIS guided fluid management can improve long term outcome in MHD patients. Body composition and hydration state will be assessed with a portable whole body bioimpedance spectroscopy device (BCM—Fresenius Medical Care D GmbH). The study will involve 16 clinical sites, enrolling more than 1300 participants. The enrolment period will last approximately 6 months, with a planned minimum 36 months follow-up period. Participants will be randomized to two arms, the control arm and BCM arm. Patients in both arms will receive BCM measurement, but results from the BCM measurement in the control group will be kept blinded to the investigators. Participants in control group will be followed up and their dry weights will be adjusted according to the dialysis center’s standard clinical practice. Clinicians managing participants in the BCM group, in addition to routine practice, will receive BCM measurement and dry weight will be adjusted according to BCM output data estimating overhydration and dry weight. All of baseline demographics, clinical data, laboratory data, regular BCM measurement, and ultrasonic cardiograph (UCG) data will be recorded on study case report forms (CRFs).

**Figure 1:**
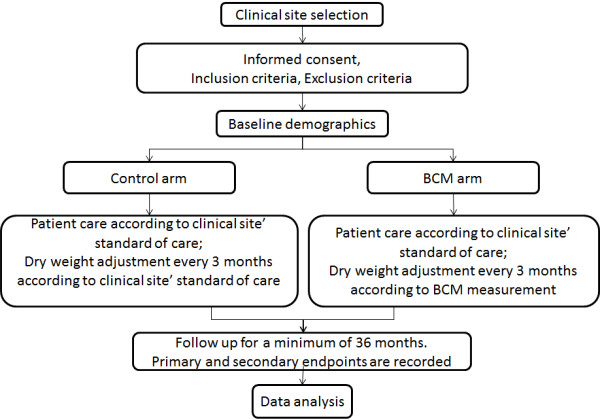
BOCOMO study Flow chart.

The flow chart of the study is shown in Figure 1.

### Study objectives

The objective of the study is to characterize the benefit of BCM guided fluid management in MHD patients.

#### Primary objective

The primary objective of the study is to compare incidence rate of a composite endpoint between BCM group and control group. Participants reach the primary endpoint if at least one of the following occurs: (1) death, (2) acute myocardial infarction, (3) cerebral thrombosis, (4) cerebral hemorrhage, (5) peripheral arterial occlusive disease. The diagnosis of endpoints will be made by the Endpoint Committee.

#### Secondary objectives

Secondary objectives are to compare the following indices between BCM arm and control arm: (1) left ventricular thickness and left ventricular ejection fraction, (2) Pre-HD and post-HD blood pressure, (3) anti-hypertensive medication, (4) hospitalization rate.

### Study protocol

#### Inclusion criteria

Candidates must meet all of the following criteria to be eligible for enrollment into the study: (1) diagnosis of end stage renal disease (ESRD) and need for MHD, (2) age of 18 to 80 years old, (3) on MHD for at least 3 months, (4) dialysis frequency of at least 5 sessions per 2 weeks, not less than 4 hours per session and Kt/V at least 1.2, (5) urine volume less than 800 mL per 24 hours the day before dialysis session, (6) BIS not used within recent 3 months, (7) dry weight regarded as adequate according to the patient’s responsible doctor, (8) the ability to understand and willingness to sign an informed consent statement.

#### Exclusion criteria

Candidates meeting any one of the following criteria will not be eligible to be included in the study: (1) acute infection within prior month, (2) active rheumatic disease, or current receipt of cortical steroid or cytotoxic medication, (3) uncontrolled neoplasm, (4) acute myocardial infarction within prior month, (5) congestive heart failure (NYHA 3 – 4), (6) stroke within 3 prior months, (7) metallic prosthesis (contraceptive device, artificial joint), (8) amputation, (9) female of childbearing age who has a pregnancy plan, or is pregnant, or is breast feeding, (10) having a plan to reduce dialysis frequency, (11) having a renal transplantation plan or planning to transfer to peritoneal dialysis within 3 years, (12) participating or planning to participate in other clinical trials which would confound the current study.

Candidates meeting inclusion and exclusion criteria will be included in the study after providing informed consent. Study approval is obtained from the Institutional Review Board, Peking University First Hospital. This study is registered on ClinicalTrial.gov and has the identification number NCT 01509937.

#### Data collection

Summary of data collection is listed in Table 1.

**Table 1:** BOCOMO study schedule of data collection

**Item**	**Baseline frequency**	**Follow up frequency**
Demographic information	Once	-
ESRD history^1^	Once	-
Comorbidity^2^	Once	-
Physical examination^3^	Once	Every 2 months
Timed urine collection^4^	Once	Every 2 months
CBC^5^	Once	Every 3 months
Routine laboratory data^6^	Once	Every 3 months
Kt/V	Once	Every 3 months
iPTH	Once	Every 3 months
hsCRP	Once	Every 3 months
UCG	Once	Every 12 months
BCM measurement	Once	Every 2 months in BCM arm
Dialysis prescription change^7^	Once	Throughout the study period
Medication change^8^	Once	Throughout the study period
New clinical event^9^	-	Throughout the study period

Abbreviation: BOCOMO study, body composition monitor study; ESRD, end stage renal disease; CBC, complete blood count; Kt/V, calculated using Daugirdas single pool equation; iPTH, intact parathyroid hormone; hsCRP, high sensitive C-reactive protein; UCG, ultrasonic cardiograph.

^1^ESRD history includes cause of ESRD, date of first dialysis, type of vascular access.

^2^Detailed history of comorbidity will be assessed, including vascular access events.

^3^Physical examination includes body weight, blood pressure, heart rate, edema, pulmonary rale, before and after hemodialysis session.

^4^24 hours urine volume collected the day before dialysis session will be recorded.

^5^Consists of hemoglobin, white blood cell count, platelet count.

^6^Consists of albumin, pre-Albumin, creatinine, urea, potassium, sodium, chloride, calcium, phosphate, bicarbonate, alkaline phosphatase (ALP), glucose, triglycerides, total cholesterol, low density lipoprotein, high density lipoprotein, brain natriuretic peptide (BNP).

^7^Any dialysis prescription change throughout the study period will be recorded.

^8^Any medication change throughout the study period will be recorded.

^9^Any new clinical event throughout the study period will be recorded, including hospitalization, new diagnosis, vascular access related event, treatment of abnormal laboratory value, dry weight adjustment.

##### Baseline data collection

In brief, baseline data are collected before initiation of BCM monitoring and include demographics, ESRD treatment history, current vascular access type, detailed comorbid conditions assessment, current dialysis prescription and medications (oral, subcutaneous and intravenous), CBC, routine laboratory data, Kt/V, iPTH and hsCRP. UCG will also be performed. BCM measurement will be done in participants in both BCM arm and control arm. In the control arm, BCM data will not be provided to patient care provider. 24 hours urine volume will be recorded the day before dialysis session. A physical examination will be performed.

##### Follow up data collection

Physical examination and urine volume collection will be performed every 2 months. CBC, routine laboratory data, and Kt/V will be obtained every 3 months. iPTH and hsCRP will be measured every 3 months. UCG will be performed every 12 months.

BCM measurement will be performed every 2 months in BCM arm, data will be recorded and provided to the care provider.

#### Dry Weight Adjustment Strategy

All participants will be educated to reduce inter-dialysis weight gain to less than 5% body weight.

For participants in the control arm, dry weight will be adjusted according to the clinical site’s standard clinical practice.

For participants in the BCM arm, dry weight will be adjusted according to the BCM estimates of overhydration and normovolemic weight. During each dialysis session, participants’ dry weight will be increased or decreased toward BCM reported dry weight. The increment or decrement of dry weight will not be greater than 0.5 Kg within a single dialysis session. Thus, in this arm participants’ dry weights will be gradually adjusted to BCM reported value. If ultrafiltration is not tolerated because of hypovolemia based on symptoms and signs, such as muscle cramps, need for excessive saline, or symptomatic hypotension, adverse events will be recorded, and the actual dry weight will be adjusted by plus 0.5 Kg. If participants experience hypervolemic clinical episodes, such as hypertension related symptom, congestive heart failure, pulmonary edema, adverse events will also be recorded. If a patient experiences the same adverse effect of dry weight change for two consecutive visits, the patient will be removed from the study because of adverse event.

**Figure 2:**
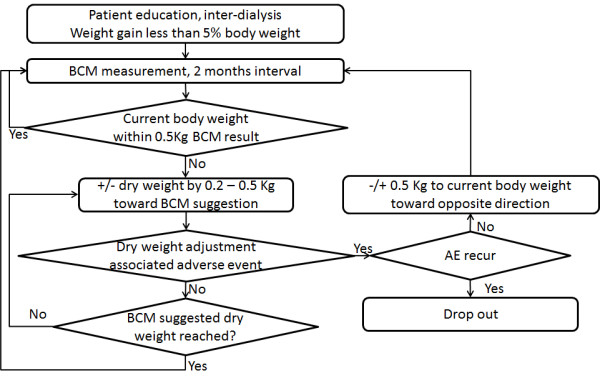
Dry weight adjustment flow chart in the BCM arm.

Dry weight adjustment flow chart in the BCM arm is shown in Figure 2.

#### Clinical events

##### Dialysis prescription change

Dialysis prescription may be changed according to clinical site’s standard practice including changes of dialyzer, blood flow rate, dialysate flow rate, frequency of dialysis, dialysis modality (conventional hemodialysis, high flux dialysis, hemofiltration, hemodiafiltration, and in combination of blood perfusion). Kt/V will remain not less than 1.2 despite dialysis prescription change. Dialysis prescription change is regarded as a clinical event and will be recorded.

##### Medications change

Medications (oral, subcutaneous and intravenous drug administration) can be changed according to clinical site’s practice, this including anti-hypertensives, active vitamin D and its analogues, erythrocyte stimulating agents (ESA), and iron supplements. Medications change is regarded as a clinical event and will be recorded.

##### New diagnosis

Any newly emerged diagnosis is regarded as a clinical event and will be recorded; hypotension and related clinical episode, hypertension and related clinical episode, pulmonary infection, diarrhea, angina pectoris, myocardial infarction, ischemic and hemorrhagic stroke, peripheral arterial occlusive disease, newly diagnosed neoplasm.

All deaths will be recorded as to date of death and cause.

##### Vascular access event

Vascular access bleeding, thrombosis or malfunction will be recorded.

##### Dry weight change

Each step of weight adjustment will be recorded.

##### Hospitalization

Hospitalization will be recorded, including diagnosis leading to hospitalization, date of hospitalization, length of stay, and outcome.

##### Abnormal laboratory value

If a procedure is performed to deal with abnormal laboratory value, the abnormal laboratory value is a clinical event and will be recorded.

##### Data censoring

The following participants are censored: (1) transfer to peritoneal dialysis, (2) renal transplantation, (3) becomes pregnant, (4) receives metal implantation, or any other reasons that make BCM measurement impossible, (5) limb amputation, (6) withdrawal of consent, (7) receives any forms of BIS measurement other than those specified by the trial protocol, (8) relocates to other clinical site, (9) investigator requests the participant be withdrawn from the study.

#### Safety issue

The clinical use of BIS method had been approved by State of Food and Drug Administration (SFDA). As one form of BIS method, BCM device is widely used in Europe. BIS inject weak alternating current into human body, which cause no harm to human body.

### Statistical analysis

#### Study hypotheses

This study is designed to test the primary hypotheses that patients receiving BCM guided fluid management have lower rate for the composite endpoint of mortality, cardiovascular and cerebrovascular events compared with patients receiving fluid management according to clinical site’s standard care. The secondary hypotheses that will be tested are that patients receiving BCM measurement have (1) lower left ventricular thickness and higher left ventricular ejection fraction, (2) better blood pressure control, (3) less anti-hypertensive medication, (4) lower hospitalization rate.

#### Sample size calculation

Literature search failed to identify a study of similar design to this one. In an observational study by Wizemann et al. [1] of 269 MHD patients the patients were divided into hypervolemic group if their ECF was 15% higher than normal or normohydrated group if less than 15%. The patients were followed for an average 3.5 years. There was 41% death in the hypervolemic group, and 30% death in normohydrated group. Relative risk of death was 36.6% higher in hypervolemic group than in the other.

According to data from Beijing Hemodialysis Quality Control and Improvement Center, the annual mortality rate of Beijing MHD patients is around 10%, it is estimated that 3 year mortality to be 30%. It is also estimated that the rate of composite endpoint within 3 year period of time is 40%. We make an assumption that BCM guided fluid management will reduce the 3-year composite endpoint rate from 40% to 32% (20% relative risk reduction). To reach statistical significance with α < 0.05 and 1-β > 80%, the sample size required is 1128. Allowing for a 20% loss-to-follow-up, the total sample size planned is 1354.

#### Analytical method

##### Primary endpoint

The primary analysis will be an intent-to-treat analysis comparing composite endpoint between the BCM and the control arms. The intent-to-treat population is defined as all enrolled study participants. For this analysis, the follow-up time begins from the enrollment date to the composite endpoint, loss to follow-up, or end of study.

Multivariate Cox proportional hazards regression models will be used for analysis of the risk of death comparing BCM and control arms, with adjustment for age, sex, dialysis vintage, primary cause of ESRD, comorbid conditions, and other characteristics that may be associated with increased risk of death, such as inter-dialysis weight gain, hypertension and hsCRP.

##### Secondary endpoint

The following 3 secondary endpoints will be compared between BCM and control arms using grouped t-test, and within group trend will also be analyzed: (1) left ventricular thickness and left ventricular ejection fraction, (2) pre-HD and post-HD blood pressure, (3) anti-hypertensive medication.

The secondary endpoint of incident rate of hospitalization will be compared between BCM arm and control arm using general Linear Model with Poisson regression.

##### Interim analysis

The final analysis for primary endpoint and secondary analysis will be performed after completion of study.

Interim analysis is planned 1 year and 2 years after study initiation. The purpose of interim analysis includes: (1) adjustment of sample size according to collected real data, (2) early termination of study if statistically significance reached comparing primary endpoint between BCM arm and control arm, (3) early termination of study if incidence rate of fluid management related adverse events is significantly high in the BCM arm.

## Discussions

### Strengths

Previous results regarding the benefit of strict fluid control are conflicting due to small sample sizes and unstable dry weight estimating methods. To our knowledge this is the first large-scale, multicentre, prospective, randomized controlled trial to assess whether BIS-guided volume management improves outcomes of MHD patients. The endpoints of the BOCOMO study are of utmost importance to health care providers.

### About the design

#### Inclusion and exclusion criteria

(1) Only MHD patients who had been on MHD for at least 3 months are eligible. This is because patients with dialysis vintage less than 3 months are more likely to be hemodynamically unstable. Additionally, this criterion reduces the likelihood that pre-ESRD factors will be introduced into the study confounding the primary result. (2) Limiting inclusion to patients with urine volume less than 800 ml/day the day before dialysis session will limit confounding due to residual renal function effects on the measured parameters. (3) Patients who had received BIS measurement within 3 months prior to enrolment are excluded as data from such measurements might lead to protocol violation.

### Changes of dialysis prescription and medications

Fluid status change will definitely lead to physiological changes of the body. It is reasonable that some clinical parameter will change, like blood pressure, hemoglobin, hsCRP, BNP. So it is reasonable to allow dialysis prescription and medications change, which will serve as one of the secondary endpoints.

### Endpoint and sample size

Annual mortality of Beijing MHD patients was around 10%. To reach statistical significance, the sample size will be very large. By using composite endpoint, the sample size becomes reasonable and feasible.

### Dry weight adjustment in BCM arm

“Real dry weight” is unknown in BOCOMO study. BCM reported dry weight might by significantly higher or lower than the real dry weight, which will lead to hypervolemia related acute symptoms and hypovolemia related acute symptoms, respectively. They will also worsen patients’ long-term outcome. In these kinds of conditions, adverse events attributes to BCM arm will be recorded.

Another issue regarding dry weight adjustment in BCM arm is the amount of each dry weight increase or decrease. Large amount of dry weight change is not allowed because significant dry weight increase or decrease during one HD session will lead to dramatic blood volume increase or decrease during HD session and associated clinical manifestation.

### Limitations

There are several limitations of the BOCOMO study: (1) using composite endpoint instead of mortality, (2) inclusion of prevalent patients instead of incident patients (but dialysis vintage will be included in multivariable analysis), (3) most clinical sites are located in Beijing.

## Conclusion

In summary, the BOCOMO study has the potential to be the largest and the first prospective randomized controlled trial to evaluate the long term clinical benefit of BIS-guided fluid management in MHD patients. The aim of the study is to validate whether BIS based dry weight adjustment decrease incidence composite endpoint of death, acute myocardial infarction, stroke, and peripheral arterial disease. The result will be of great clinical importance.

## Competing interests

The authors declare that they have no competing interests.

## Authors’ contributions

All authors participated in the design and discussion of the protocol. All authors read and approved the final manuscript.

## Pre-publication history

The pre-publication history for this paper can be accessed here:

http://www.biomedcentral.com/1471-2369/13/120/prepub
